# A fast region-based active contour for non-rigid object tracking and its shape retrieval

**DOI:** 10.7717/peerj-cs.373

**Published:** 2021-05-27

**Authors:** Hiren Mewada, Jawad F. Al-Asad, Amit Patel, Jitendra Chaudhari, Keyur Mahant, Alpesh Vala

**Affiliations:** 1 Electrical Engineering, Prince Mohammad Bin Fahd University, Al Khobar, Kingdom of Saudi Arabia; 2 CHARUSAT Space Research & Technology Center, Charotar University of Science and Technology, Changa, Gujarat, India

**Keywords:** Active contour, Computer vision, Image segmentation, Mean-shift tracking

## Abstract

Conventional tracking approaches track objects using a rectangle bounding box. Gait, gesture and many medical analyses require non-rigid shape extraction. A non-rigid object tracking is more difficult because it needs more accurate object shape and background separation in contrast to rigid bounding boxes. Active contour plays a vital role in the retrieval of image shape. However, the large computation time involved in contour tracing makes its use challenging in video processing. This paper proposes a new formation of the region-based active contour model (ACM) using a mean-shift tracker for video object tracking and its shape retrieval. The removal of re-initialization and fast deformation of the contour is proposed to retrieve the shape of the desired object. A contour model is further modified using a mean-shift tracker to track and retrieve shape simultaneously. The experimental results and their comparative analysis concludes that the proposed contour-based tracking succeed to track and retrieve the shape of the object with 71.86% accuracy. The contour-based mean-shift tracker resolves the scale-orientation selection problem in non-rigid object tracking, and resolves the weakness of the erroneous localization of the object in the frame by the tracker.

## Introduction

The captured video contains moving objects and sounds representing a real-life scene. It gathers the sequence of images and plays them continuously. The human eye is sensitive to watch these sequences as a video if at least 24 sequences are played per unit second (Larson, 2020). In the tracking process, a trajectory of the desired object is estimated in the space domain as it moves from one frame to another frame. Thus it is the process of assigning a particular label in each frame. According to the application requirements, object-centric information like its shape, orientation, area, and recognition can be obtained after tracking. Visual object tracking (VOT) is a challenging task due to many issues like poor quality of the camera (i.e. low resolution, color discrimination, low frame rate), other environmental challenges like the small size of the object, clutter background, non-rigid shape, occlusion, appearance variation and lastly real-time tracking requirement. To target these challenges, various object appearance forms have been used in VOT. These tracking forms are illustrated in Li et al. (2013).

Major VOT literature implements a robust tracking algorithm for the aforementioned challenges using either rectangular bounding box or ellipse. A contour-based tracking referred to as non-rigid object tracking is more challenging because it needs more accurate object shape and background separation in contrast to rigid bounding boxes. Non-rigid object tracking and its analysis have broad applications in real-life, i.e. gait analysis and human recognition, automated object classification, detection of suspicious activities in an automated surveillance application, annotation of retrieval of a particular video from the dataset in video indexing application, traffic monitoring, tracking in medical imaging for clinical diagnosis, gesture detection and tracking for robotic applications and so on.

The non-rigid tracking algorithms generally rely on local pixel information (Duffner & Garcia, 2013), superpixel (Wang, Lu & Yang, 2017), or patch-based segmentation approach (Godec, Roth & Bischof, 2013). In local pixel information, object’s pixels are localized and discriminated from the background using a Hough voting scheme and probabilistic segmentation algorithm. In superpixel, a confidence map is obtained from using a discriminative algorithm to segment and track the object. Whereas, in patch-based segmentation, a Hough forest voting is used to classify the patches of the desired object from the frame. A radon transform features from the images were extracted to identify non-rigid patterns from the images in Santosh, Lamiroy & Wendling (2011, 2013). They used histogram at various projection angle in the Radon domain. To avoid the problem of compressed representation due to a single vector formation of the histogram, the authors proposed a dynamic time wrapping method. They found that this method is more robust to is deformed shape due to degradation, distortion and occlusion. These algorithms use handcrafted features, thus limiting the accuracy for the complex object. Besides, there is no correlation between the tracking and detection process.

Active contour has played a significant role in non-rigid image segmentation and it has been widely used in medical image segmentation (Beaupré, Bilodeau & Saunier, 2018; Mewada et al., 2020) e.g. tissue shape extraction, tumor detection, aerial, and natural image segmentation (Patel, Mewada & Patnaik, 2012), and texture image segmentation (Mewada, Patel & Patnaik, 2015). The energy calculation based deforming nature of contour in an image plane can well extract the shape of the object automatically. Therefore, this paper proposes a non-rigid object tracking using the active contour model. The major challenge in contour-based tracking is the computation time. Hence, a concurrent approach for non-rigid object tracking and its shape extraction using active contour model and mean shift tracking algorithm is proposed. The overall structure of the paper is as follows: “Related Work” classifies the tracking algorithms and then presents the study of contour-based tracking algorithms. “Proposed Non-Rigid Object Tracking Algorithm” introduces fast active contour model initially and later the proposed concurrent approach is explained in detail. “Results and Discussion” discusses the obtained results and their analysis. In the end, a conclusion and future scope are presented in “Conclusion”.

## Related work

The three crucial steps involved in the tracking process of moving objects in a video are: (a) detection of the desired object (b) motion tracking of that object and (c) behavioural analysis or monitoring of the object to fulfil a particular task. The detection of the object requires a mechanism either in all sequences or in one particular frame of the sequences referred to as a reference frame. Based on the detection approach, the tracking process can be classified as detection inclusive or detection free tracking. The use of a particular object detection algorithm and its linking to trajectories in the tracking process (Bose, Wang & Grimson, 2007; Yu & Medioni, 2008) is referred to as detection inclusive tracking whereas, manual initialization of object in the first frame and localizing it in subsequent frames is referred to as detection free tracking (Hu et al., 2012; Yang, Lu & Yang, 2014; Zhang & Van der Maaten, 2013). Detection free tracking is the more efficient however it requires user interaction and thus it is a semi-automatic method.

The most commonly used detection mechanisms are background subtraction, static approach, time difference, and optical flow (Yang, Shi & Yi, 2012). In optical flow (Mahraz, Riffi & Tairi, 2014; Beaupré, Bilodeau & Saunier, 2018), the motion vectors obtained from the features set of the object to be tracked are passed through the optical flow function for each frame. The motion vector calculation requires patches of the images and hence it is a time-consuming process causing difficulties in real-time implementation. Time difference mechanism finds the motion regions and calculates the difference between two consecutive frames’ pixels. It is mostly adaptive but erroneous in the detection. In the static approach, a reference frame not containing moving objects i.e. static background is used. All pixels in the current frame are modeled by Gaussian distribution function (GDF) by finding the maximum intensity difference between these two frames. The major constraint of this method is the requirement of the background frame. In the background subtraction process, pixels are modeled by Gaussian mixture model (GMM) using few initial frames to find the foreground object. Later, pixels are processed using morphological operation and thresholding to find actual objects (Israni & Mewada, 2018b).

Object tracking methods can be categorized based on utilization of the features and approaches used for the detection. In features based VOT, various features of the object like color (Aoki, Oki & Miyamoto, 2020), texture (Khilar et al., 2020), edges, and statistical features (Israni & Mewada, 2018a) are used to track the object. Detection based tracking algorithms can be classified further as point tracking, kernel tracking, Silhouette/contour tracking, and supervised learning approach. The tracking using supervised learning requires pre-training of the algorithm and hence it is largely used for the online tracking process. Point tracking is robust to the illumination but it requires an additional mechanism to find the object in each frame. The kernel-based tracking is poor to track a non-rigid object in the video. Silhouette/contour-based tracking can handle a large variety of shapes of the desired object.

Major tracking algorithms have used edge-based active contour (i.e. snake) (Leymarie & Levine, 1993), active meshes (Valette, Magnin & Prost, 2001), or region-based active contour (Pateux, 2000). A kernel and contour-based hybrid model was presented in (Yilmaz, 2007), where authors generated the kernel using implicit contour and estimated the object’s new location with its scale and orientation. Yi, Ahn & Choi (2008) used a mean shift algorithm in the tracking process and presented a probabilistic approach to estimate the orientation and scale of the object. The use of the fixed shape kernel failed to detect the shape when the object rotates out of the plane. Nakhmani & Tannenbaum (2012) eliminated the false loop generation in parametric contour and used the parametric contour to track the human under noisy environment.

Silhouette based shape representation and tracking of moving object were presented by Toshev, Makadia & Daniilidis (2009). They used the Kanade-Lucas-Tomasi (KLT) tracker to extract the features and track the object. Using the obtained track labels, they segmented the object and obtained its silhouettes. They evaluated their model on a dataset containing a car, airplane, and helicopter. In Cremers (2006), tracking of multiple objects using posterior probability framework was proposed and the shapes of objects were retrieved using the level-set. Non-rigid object tracking in video captured by moving camera was proposed in Lefèvre & Vincent (2004). They used edge-based ACM to track and segment the object. To achieve real-time performance on outdoor scene video sequences, the gradient information was computed in the region of the object only once per frame. To quantify animal behaviour, a body and leg tracking process of Drosophila was presented by Uhlmann et al. (2017). A semi-automatic active contour-based tracking method reduced the user intervention and opened a new direction to annotate the video of species. An optical flow based background modelling and car’s shape extraction and its classification using hidden markov model (HMM) were presented in Thakoor & Gao (2005). The feature set including color and texture was integrated to level set and proposed in Xian, Hu & Zheng (2013) to extract non-rigid shape for surveillance dataset.

Baum (2013) utilized depth images along with color information of the object to track it. By integrating the depth of the object, the shape of the object was generated. Lucas-Kanade algorithm was used in optical flow measurement and the Generalized Probabilistic Descent model was used with HMM. A scale and orientation adaptive kernel, driven through an active contour model was proposed by Sun et al. (2020). They evaluated the algorithm on VOT2014 and VOT2016 dataset achieving an average accuracy of 65.93% and 54.53% respectively. A fragment-based object tracking in the contour framework was proposed in Chockalingam, Pradeep & Birchfield (2009). A saliency map generation using Deep Neural network for object tracking and shape extraction was proposed in Zhang et al. (2020). This supervised algorithm trained for one dataset is not applicable to another dataset unless the network is re-trained. In summary, a very limited tracking algorithm was proposed which provides the shape of the tracking object. The draw back of this algorithm is represented in its high computational cost and limited accuracy and that is in comparison with bounding box based tracing algorithm.

A sparse representation can reduce the data vector size and increase the speed of execution (Patel & Mewada, 2018). A sparse dictionary formation and visual object tracking was presented in Joshi & Mewada (2020). Multiple patches are extracted from each frame to form the sparse dictionary, and likelihood measurement is carried out to track singular object handling long term occlusion. They conclude that patch wise joint sparse tracking produced better results under various constraints of occlusion, illumination variation, deformation and blurring. Automated tracking using Gaussian Mixture Model (GMM) in real-time for the robotic application was presented in Patoliya & Mewada (2019). They achieved 85% tracking accuracy in the ROS environment. Supervised non-rigid object tracking algorithms using RGB-depth sensors are also proposed in many literatures. A convolutional neural network based learnable approach for non-rigid tracking was proposed in Bozic et al. (2020). They used RGB-depth images and predicted dense corresponds for tracking the object. Sengupta (2020) reduced a combination of photometric and depth error and presented tracking of the deformable object using RGB-depth camera on both synthetic and real data. They used visual information from the RGB-depth camera and evolved the contour by calculating the contour forces. Li et al. (2020) generated pre-conditioner from a large number of training samples and proposed conditionNet in their process and increased the accuracy and speed of non-rigid RGB-depth object tracking process employing a learnable optimization process.

To track the vehicles and get information about the vehicles in a complex environment, a mean-shift and active contour-based tracking algorithm was proposed in Cai et al. (2017). Similarly, a contour of the face region is tracked by Zhou et al. (2013) using the Gradient Vector Flow (GVF) contour model and mean-shift algorithm. They proposed the tracking even under partial occlusion and full occlusion scenarios. The proposed algorithm is similar to the non-rigid tracking algorithm proposed in Sun et al. (2020), but it also uses a mean-shift algorithm for tracking and region-based ACM for shape extraction. The main objective of the proposed model is to reduce the computational complexity of ACM. Therefore, a region-based contour model with fast contour topology adoption was integrated with the mean-shift algorithm in contrast to modifying the kernel of the mean-shift algorithm used in Sun et al. (2020).

## Proposed non-rigid object tracking algorithm

**Figure 1:**
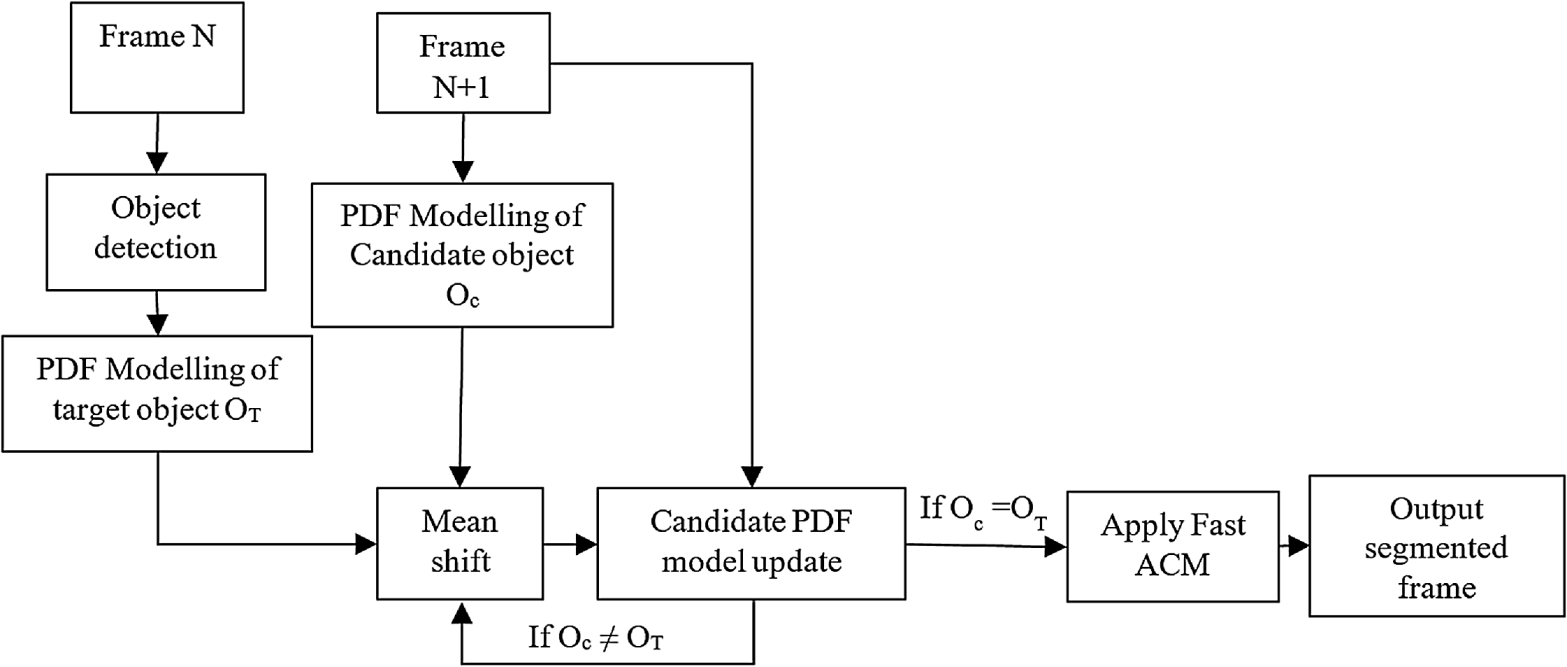
Functional block diagram of the region-based active contour model using a mean-shift tracker.

As discussed in previous section, many algorithms were proposed using a bounding box with high tracking accuracy. The classification of algorithms was also presented in “Related Work”. Among them, and as a kind of Kernel-based tracking algorithm, the mean shift tracking algorithm is the most popular due to its simplicity and tracking efficiency. The block diagram of the proposed hybrid contours based mean-shift algorithm is shown in Fig. 1.

As shown in Fig. 1A fast region-based active contour model is used to obtain the contour of the desired object. A color probability density function (PDF) is used as a feature of the object to be tracked in the proposed algorithm. The object in the first frame is selected manually. The shift of the target in the next frame is calculated using the mean-shift procedure. The last contour shape is translated to this new position in the next frame. The contour evolves in the next frame to obtain the shape of the object. The process is repeated for all frames.

### Fast active contour

The level set method is largely utilized in image segmentation applications due to its capability to handle topological changes automatically. It is a deformable contour that evolves based on applied certain energies. A Region-based ACM using level set method was initially proposed by Chan & Vese (2001). This piecewise approximated region-based ACM on image I(x,y) over the image plane *ω* is defined by the energy function i.e. *E*_*CV*_ in (1). The first two terms represent the regularization term and referred to as length and area of the closed contour (C). These two terms control the smoothness of the contour and propagation speed of the evolving contour respectively.

(1) $$\begin{array}{l} \begin{array}{ll} E_{CV}(m_{1},m_{2},\phi )= & \int_{\Omega }\int ||I(x,y)-m_{1}|{|}^{2}H(\phi (x,y))dxdy\\ & +\int_{\Omega }\int ||I(x,y)-m_{2}|{|}^{2}H(1-\phi (x,y))dxdy \end{array}\\ \begin{array}{ll} & +\mu \int_{\Omega }\int \delta_{\varepsilon }(\phi (x,y))|\nabla (\phi (x,y)|dxdy+v\int_{\Omega }\int H_{\varepsilon }(\phi (x,y))dxdy \end{array} \end{array}$$

where, *m*_1_ and *m*_2_ are the mean intensity values inside and outside the contour and *ϕ* is signed distance function. *H*_*ε*_(*z*) and *δ*_*ε*_ are slightly regularized version of Heaviside function (H) and dirac *δ* function as ε→0, defined as follow.

(2) $$H(z)=(\begin{array}{cc} 1, & ifz\geq 0\\ 0, & ifz\leq 0 \end{array}\;\;\;\;\delta (z)=\frac{d}{dz}H(z)$$

The minimization of these energies pushes the contour to a desired boundary of the object. These energies can be minimized by calculating the first derivative of the level-set function as follows:

(3) $$\frac{\partial \phi }{\partial t}=\delta_{\varepsilon }\phi \left[\mu K-v-\lambda_{1}{\left(I-m_{1}\right)}^{2}+\lambda_{2}{\left(I-m_{2}\right)}^{2}\right]$$

**Figure 2:**
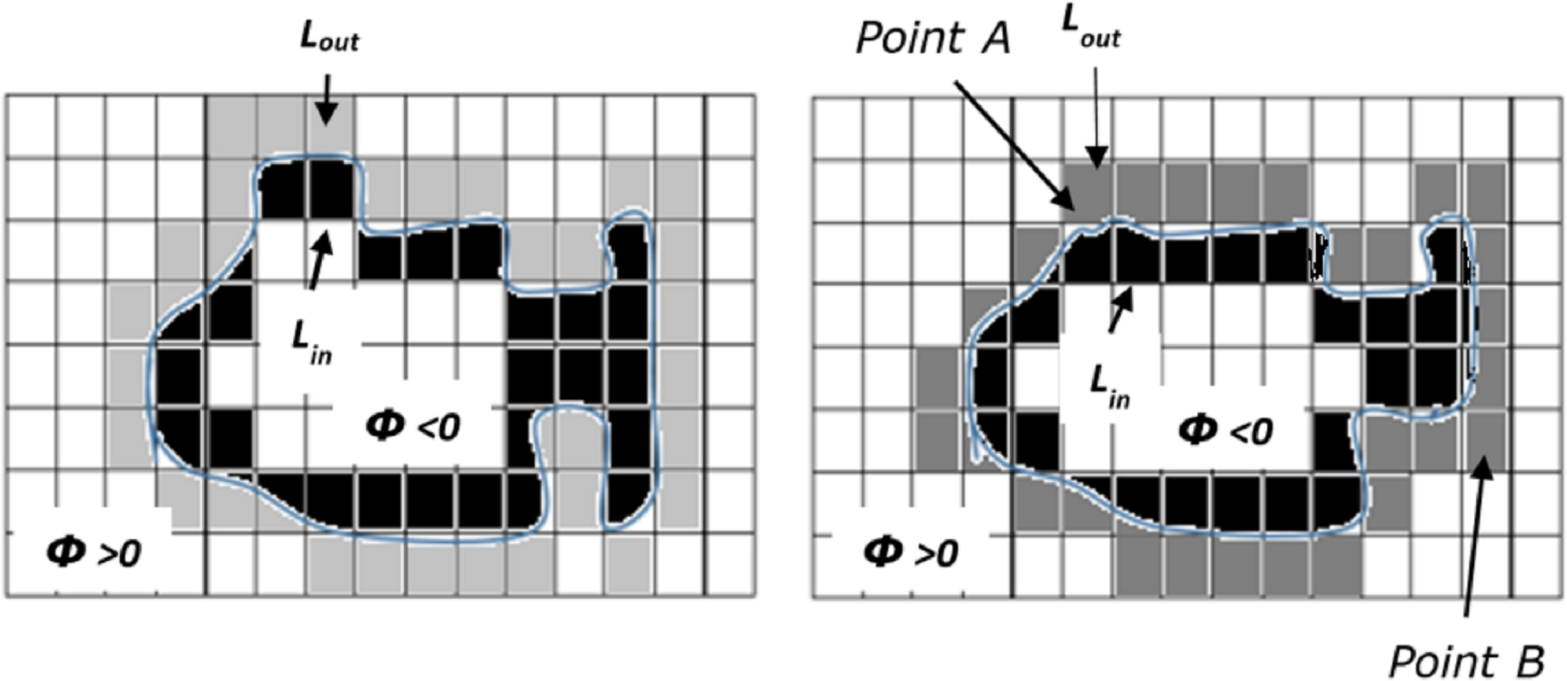
Evolution of contour (adopted from Shi & Karl, 2008).

Where, $K=div\left(\frac{\nabla \phi }{|\nabla \phi |}\right)$ is the Kappa function representing Euclidean curvature of the contour *C*, and *λ* is a positive constant. Figure 2 shows the implied representation of the contour *C* using the level-set function. The contour deforms by moving the contour point either inside or outside the shape based on the minimization of the energy function.

To prevent the contour being too at or steep near the interface, a level is generally initialized as signed distance function (SDF) in the kappa equation, i.e. on the contour; all values of the point are zero, outside the contour; all values are positive constant and inside the contour; all values are negative constant as shown in the Fig. 2. However, the re-initialization of the contour shifts the zero level-set function away from its present boundary. The suggested solutions were to incorporate partial differential equations (Sussman & Fatemi, 1999) and penalty terms (Li et al., 2005) in the length calculation of the contour. However, re-initialization is the most time-consuming process in the level-set function, i.e. 90% of the time in contour evaluation is spared to re-initialize and to regularize the contour (Patel, Mewada & Patnaik, 2012) and hence re-initialization cannot be used in video tracking applications. Patel, Mewada & Patnaik (2012) used the Gaussian smoothing function and binarization process by eliminating the re-initialization used in the Kappa equation. Thus they modified the evaluation of the contour using the following minimization function.

(4) $$\frac{\partial \phi }{\partial t}=\delta_{\varepsilon }\phi \left[\lambda_{1}{\left(I-m_{1}\right)}^{2}+\lambda_{2}{\left(I-m_{2}\right)}^{2}\right]$$

**Figure 3:**
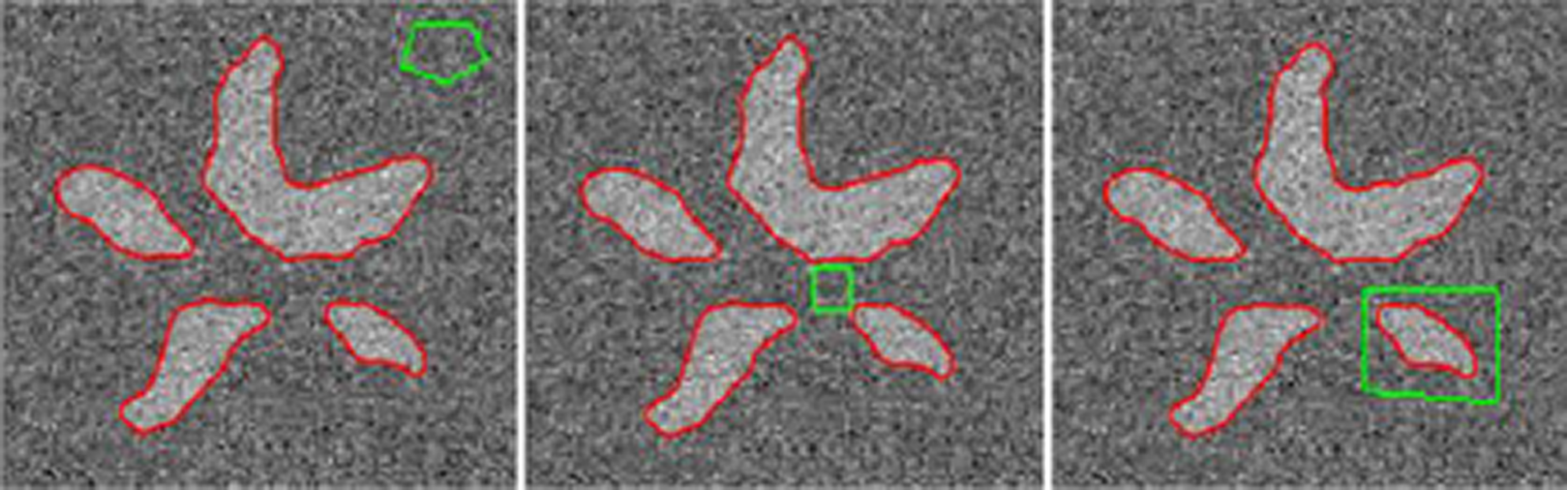
Placement insensitive shape extraction of the object (adopted from Patel, Mewada & Patnaik, 2012).

Another weakness of the standard region-based ACM is that resultant contour is highly sensitive to the placement of the initial contour in an image. In contrast to regular region-based ACM, the fast region-based ACM extracts the object boundary accurately irrespective of the placement of contour as shown in Fig. 3. This helps to track the object accurately even when the mean-shift tracker fails to locate the object. Therefore, in this paper, we used this solution to avoid the regularization and we modified the active contour to using the mean-shift tracking method to track the object and extract the non-rigid object’s shape.

### A fast region-based active contour using mean-shift tracking

In the mean-shift tracking method, a mean of the cluster is calculated and this mean is shifted to a new location in the next frame by finding a similar mean in the next frame. An object is selected using a rectangle bounding box in the reference frame. Let *x*_*i*_ where, *i* = 1,2,….,*n* , represents the pixel location of the object in the reference frame and the object is modelled using the histogram of the color of the object, then the probability (i.e. *q*) of the color (i.e. *c*) can be represented as

(5) $$q_{c}=\frac{\sum_{i=1}^{n}exp\left(\frac{-1}{2}||x_{i}{||}^{2}\right)\delta (b(x_{i})-c)}{\sum_{i=1}^{n}exp\left(\frac{-1}{2}||x_{i}{||}^{2}\right)}$$

Where *b* is histogram bin and *δ* is Kronecker delta function. If target object in the next frame is centred at *y*_*i*_, then the probability (*p*) of the color (*c*) in target object can be represented as

(6) $$p_{c}(y)=\frac{\sum_{i=1}^{n}exp\left(\frac{-1}{2}||\frac{y-y_{i}}{h}{||}^{2}\right)\delta (b(x_{i})-c)}{\sum_{i=1}^{n}exp\left(\frac{-1}{2}|||\frac{y-y_{i}}{h}{||}^{2}\right)}$$

where h is the scale where points xi are considered for probability estimation. Thus, object is localized in the next frame by finding the similarity between the models in Eqs. (5) and (6). The similarity is calculated using Bhattacharya coefficient. Using these color density models, the weight matrix at pixel (*x*, *y*) can be represented as

(7) $$W(x,y)=\sqrt{\frac{q_{c}}{p_{c}(y)}}$$

The large value of the weight W represents that the obtained model belongs to the target object model and vice versa. Therefore, the weight *W* is incorporated into region-based active contour model to obtain the non-rigid shape of the object. Therefore, Eq. (1) can be modified to look for object in the frame as follows:

(8) $$\begin{array}{l} \begin{array}{cc} E_{CV}(m_{1},m_{2},\phi )= & \int_{\Omega }\int ||W(x,y)-m_{1}|{|}^{2}H(\phi (x,y))dxdy\\ & +\int_{\Omega }\int ||W(x,y)-m_{2}|{|}^{2}H(1-\phi (x,y))dxdy \end{array}\\ \begin{array}{cc} & +\mu \int_{\Omega }\int \delta_{\varepsilon }(\phi (x,y))|\nabla (\phi (x,y)|dxdy+v\int_{\Omega }\int H_{\varepsilon }(\phi (x,y))dxdy \end{array} \end{array}$$

Where, $m_{1}=\frac{\int_{\Omega }\int W(x,y)H(\phi (x,y))dxdy}{\int_{\Omega }\int H(\phi (x,y))dxdy}$ and $m_{2}=\frac{\int_{\Omega }\int W(x,y)(1-H(\phi (x,y)))dxdy}{\int_{\Omega }\int (1-H(\phi (x,y)))dxdy}$

The minimization of the energy function in Eq. (8) gives the final position of the contour over the object boundary and is given by

(9) $$\frac{\partial \phi }{\partial t}=\delta_{\varepsilon }\phi \left[\lambda_{1}{\left(W(x,y)-m_{1}\right)}^{2}+\lambda_{2}{\left(W(x,y)-m_{2}\right)}^{2}\right]$$

Therefore, the proposed model which integrates the mean-shift algorithm with ACM is having a fast evaluation in comparison with the model proposed in Sun et al. (2020).

## Results and discussion

**Figure 4:**
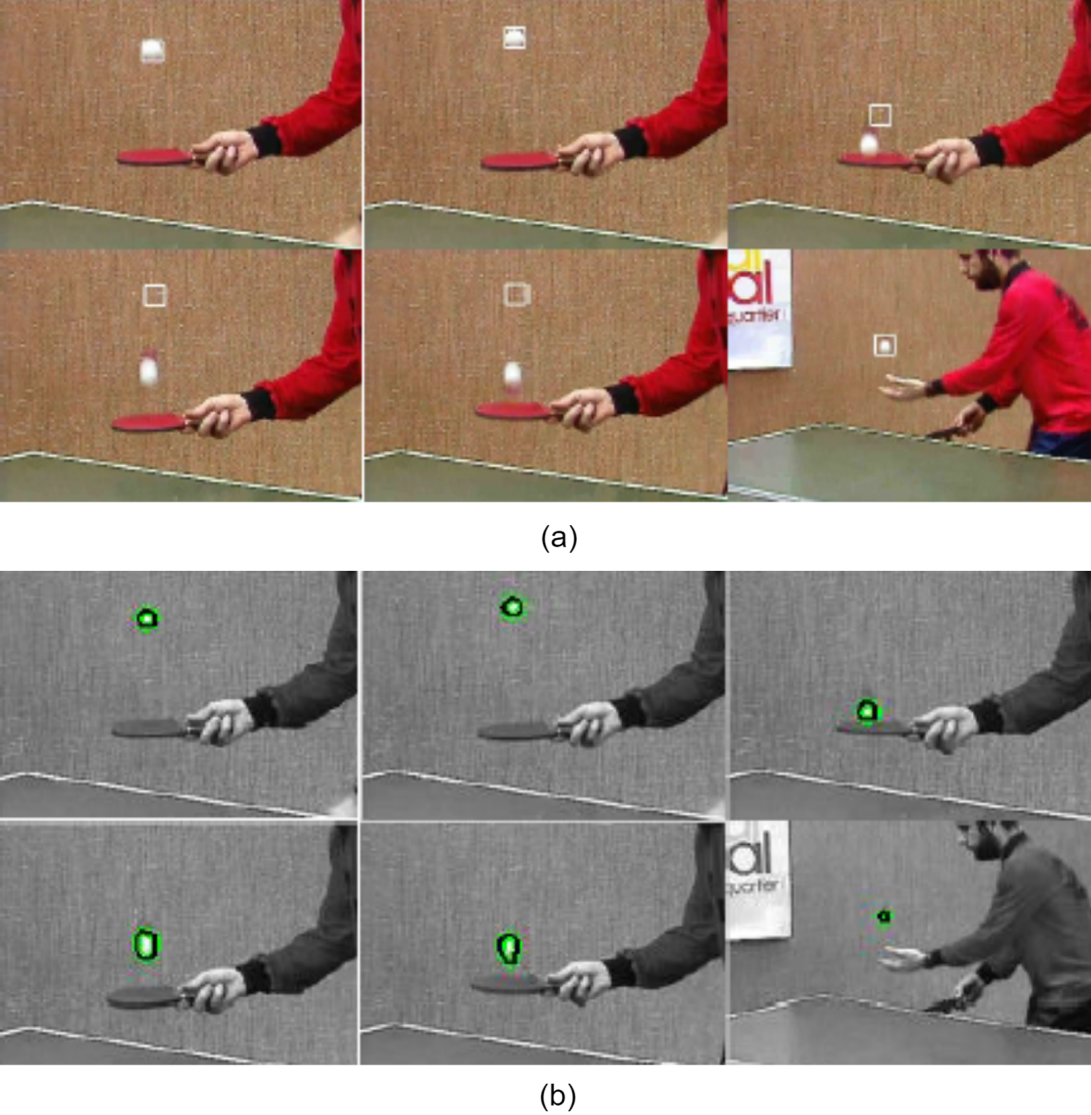
Tracking result of table tennis ball sequences (A) using mean-shift tracker (B) using proposed approach (Frames 1, 2, 11, 25, 27, 52 are displayed from left to right). Image credit: https://media.xiph.org/video/derf/.

This section analyses quantitatively the results obtained through the proposed model using various video datasets. The proposed algorithm is designed in MATLAB V2020, installed on Pentium I7 processor with 8GB RAM, 1.90 GHz PC. The proposed algorithm is also tested for real-time videos. In all cases, the color model with 16 bins is used as a feature space in the mean-shift model. Initially, a rectangle bounding box is set manually. The first experimental result of table tennis video is shown in the Fig. 4. This video consists of 52 frames sequence with a resolution of 352 × 240. The frame rate is 15 frames/second. These are the basic steps of object tracking in real-time video using a mean shift algorithm.

In Fig. 4A, top left frame shows an initial frame of the video. Taking this as a reference frame, a square mask is created on it. In all frames, the object is tracked in rectangle throughout the video as shown in following images. As shown in Fig. 4, the mean-shift cannot track objects when the frame goes relatively fast and the object is moving fast in that frame. In Fig. 4B, proposed ACM model using mean-shift tracker is depicted. As explained in “Related Work”, mean shift tracker may fail to localize the object in the next frame and hence it fails to track object. However, the contour can shift its position by minimizing the energy function and locate the object in the deformation process irrespective of its placement carried out by the mean-shift tracker. Therefore, it tracks object correctly with retrieval of the shape as shown in Fig. 4B.

**Figure 5:**
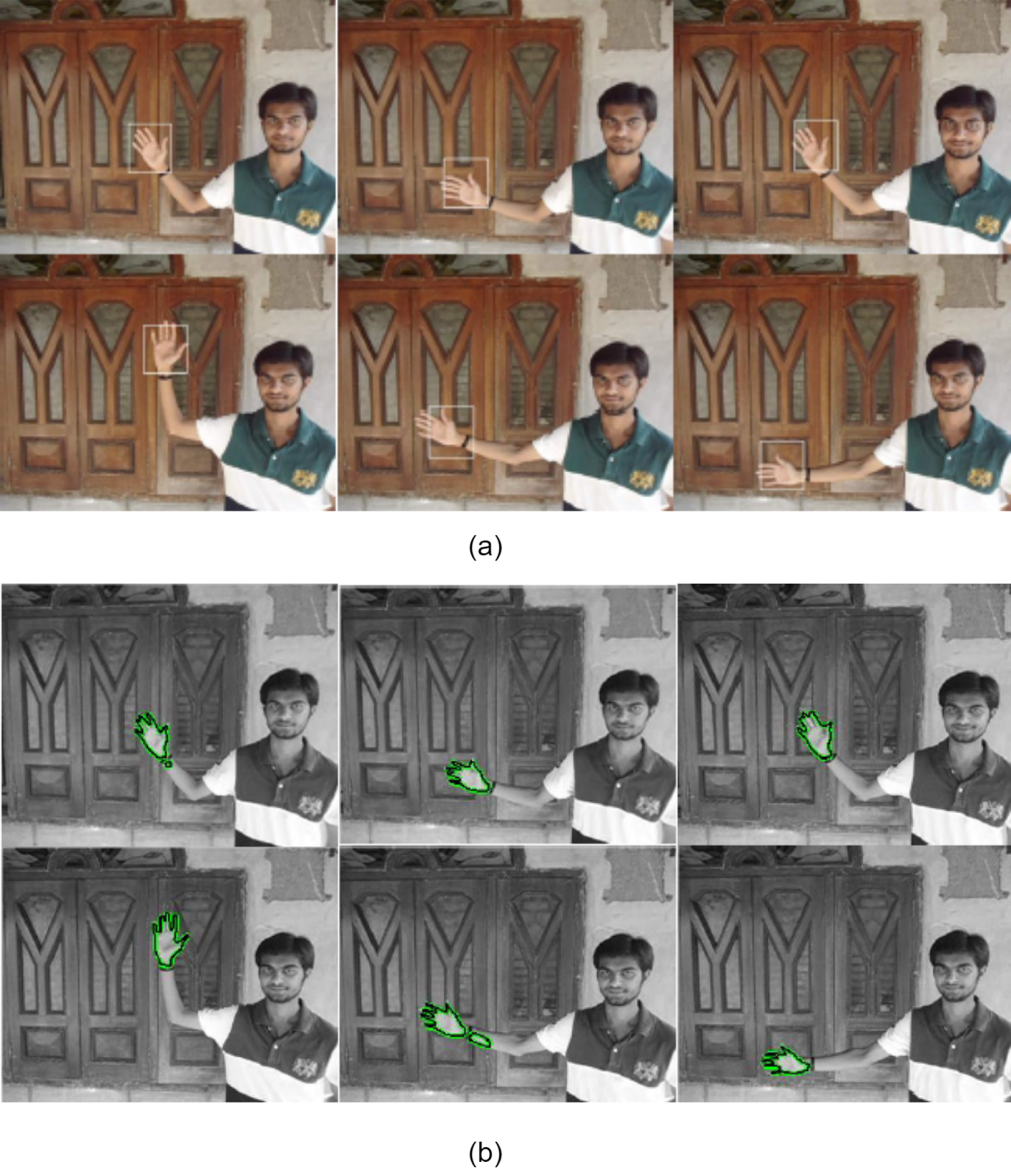
Tracking result of hand sequences with target representation models (A) using mean-shift tracking (B) using proposed algorithm. (Frames 1, 29, 100, 163, 179, 185 are displayed from left to right).

Another experiment was done on real-time video with complex background to validate processing capability of fast ACM. Figure 5 shows the hand tracking in real-time video captured using the standard camera where background contains a variety of shapes. The visual results of the mean-shift and proposed model show that the proposed model gives robust and accurate tracking of the hand object. In some frames of video, the rectangle window obtained using mean-shift is not covering the hand region from the centre of the bounding box. The proposed model gives correct tracking of the actual shape of the object in most of the frames throughout the video. Other experimental analysis was obtained for the video containing slow and rapid movement of the object.

**Figure 6:**
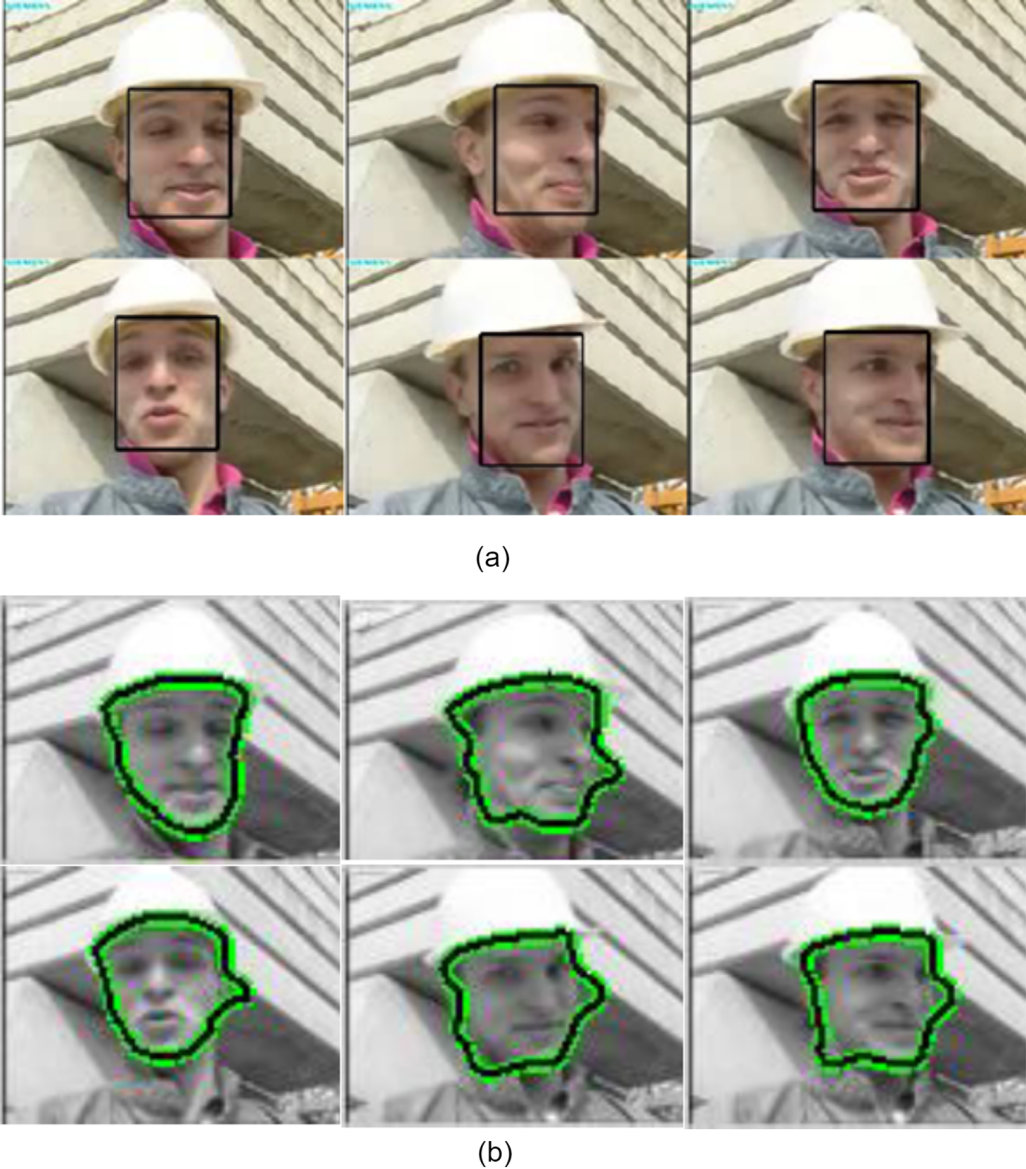
Tracking result of foreman sequences (A) using mean-shift tracking (B) using proposed algorithm (Frames 1, 6, 51, 84, 114, 126 are displayed). Image credit: Li et al. (2020).

Figure 6 shows a video clip of foreman with warped background and moving his head dynamically. At the end of video, the movement is very rapid. The warped background also creates the blocking artefacts and hence contour may fail to retrieve the desired boundary from of the tracking object. The object’s boundary is degraded at some point but mean-shift tracker assist contour to stick with defined boundary and hence it extracts the shape precisely.

**Figure 7:**
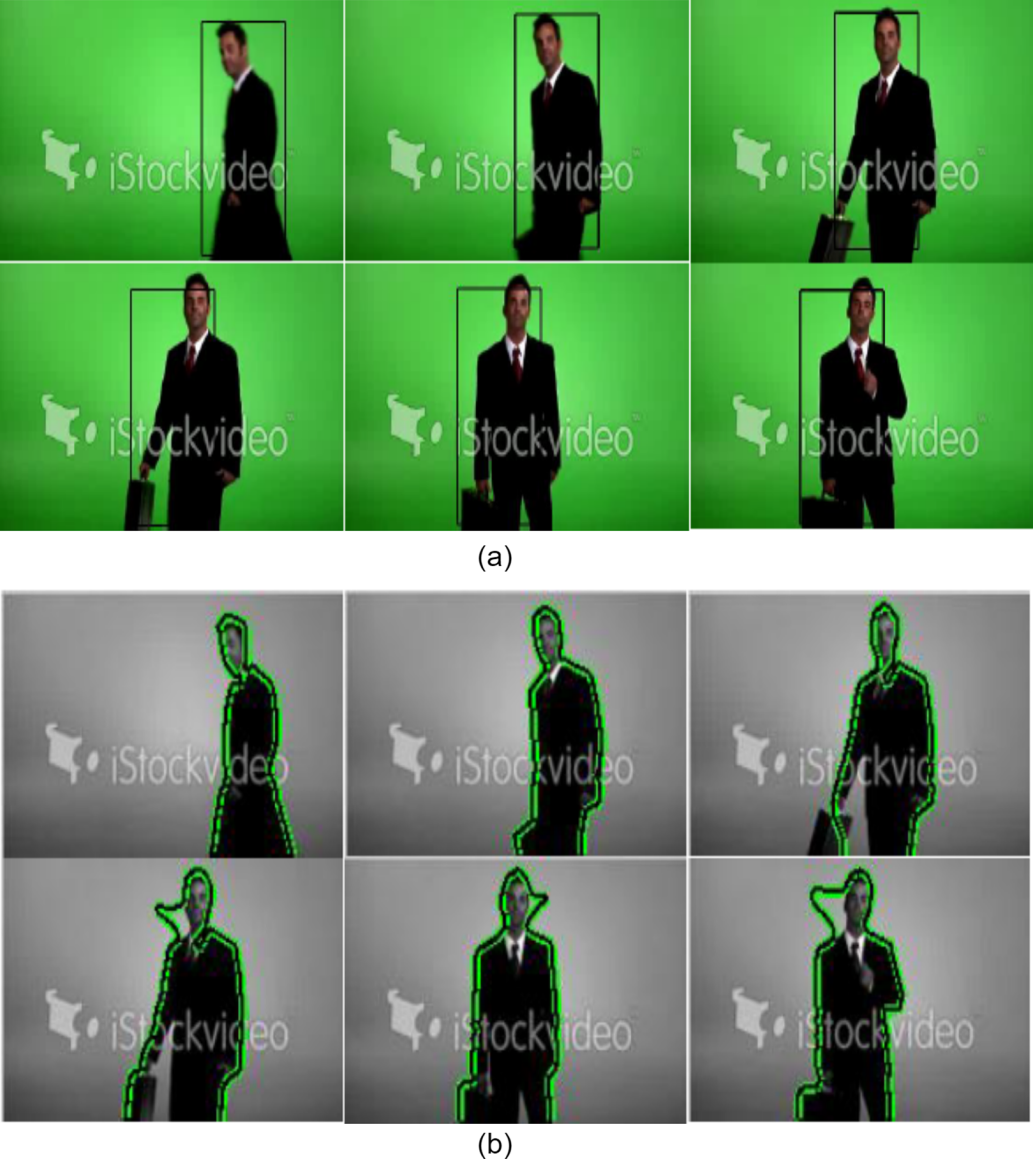
Tracking result of man sequences (A) using mean-shift tracking (B) using proposed algorithm (Frames 40, 50, 64, 69, 95, 130 are displayed). Image credit: © iStockvideo.

Here another experimental result is shown in Fig. 7, for a man tracking video containing 91 frames sequence with a resolution of 380 × 216. The frame rate is 30 frames/second. A tracking target is a man with a briefcase. Here in this video sequence, the proposed model succeeds to follow the boundary of the man in all sequences. In some of the frames, the contour did not evolve completely (i.e. near the face region in the frame 69, 95 and 130) because the number of iterations used in the contour evaluation is not competitive enough. Large number of iterations is required due to large object’s size.

**Figure 8:**
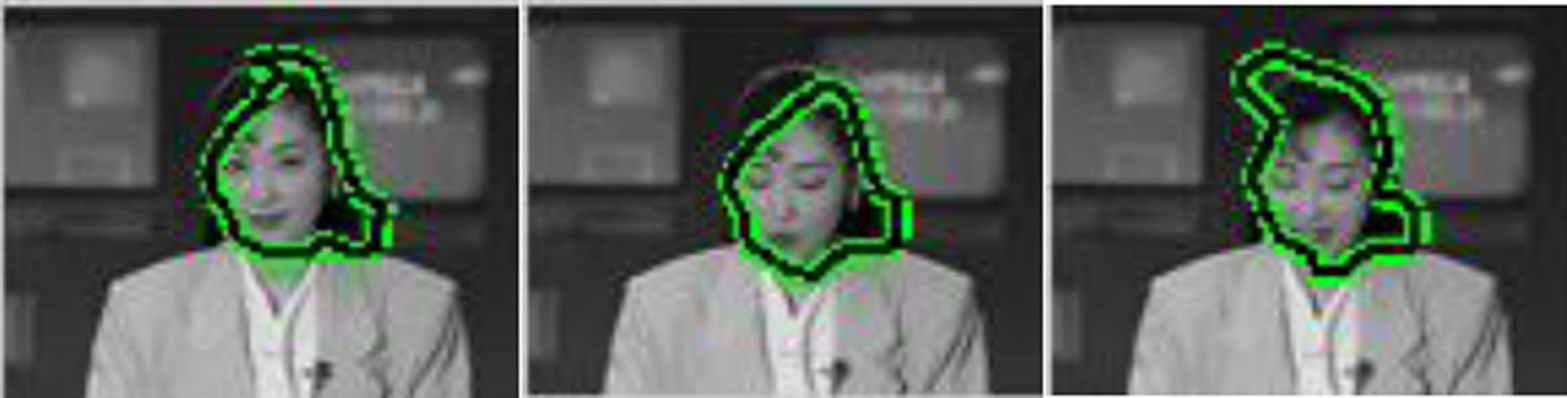
Tracking result of Akiyo sequences with target representation models (Frames 1, 120, 161, 210, 250 and 287 are displayed). Image credit: https://media.xiph.org/video/derf/.

The last video sequence is of news reader, Akiyo. It contains 300 frames with 192 × 144 resolution and a frame rate of 30 frames/second. The tracking target is the face of Akiyo whereas the moving objects are lips and eyes only. Here, the face is an object to detect and track. The result of the proposed model is shown in the Fig. 8. It is visible that at some instant the result is not accurate. This is due to background matches with the hair of Akiyo and also a television in the background is a non-targeted object for contour detection. Else overall, tracking and detection are good and accurate.

**Figure 9:**
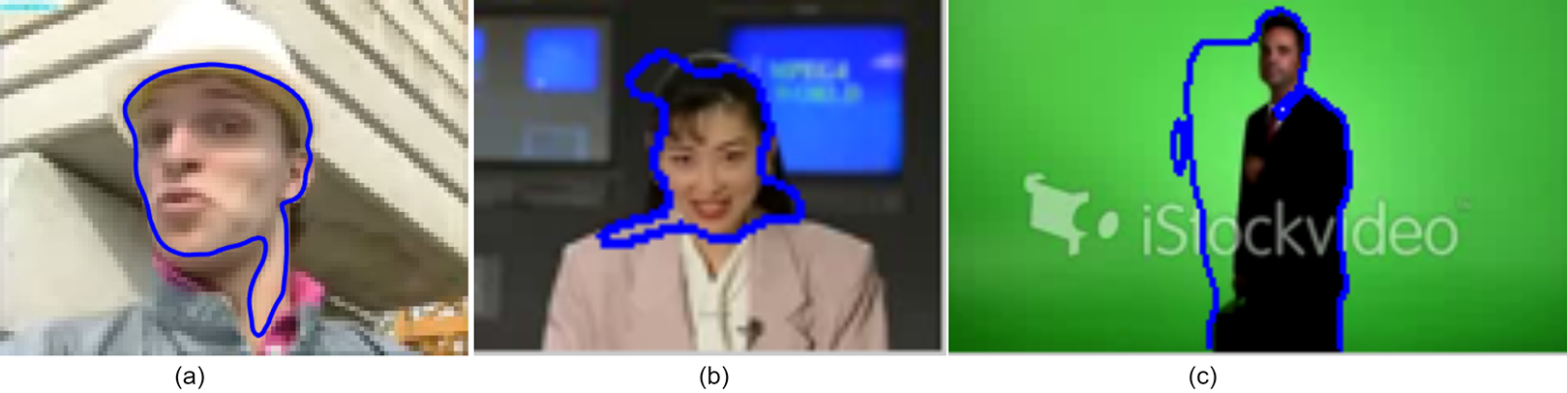
Illustration of miss-segmentation (A) Frame:155 from foreman (B) Frame:262 from Akiyo (C) Frame: 74 from man sequences. Image credit: © iStockvideo; Li et al. (2020); https://media.xiph.org/video/derf/.

Few frames from the different videos, where the algorithm can track the object correctly but not able to segment accurately are shown in Fig. 9. In the first two figures, the initialized contour has more aspect ratio than the desired object’s aspect ratio. Therefore, the assigned number of the iteration is less to evolve the contour over the desired boundary. In 155^*th*^ frame of foreman sequences, the face is occluded by the hand, and hence contour fails to grow over the face region.

Table 1 shows the quantitative analysis of all videos. Table 1 proposes that the reduction of the computation complexity in a fast-region based ACM model allows the mean-shift tracker to extract the shape of the object even in 30 frames/second videos. Further the failure of tracking due to mean- shift algorithm is also handled by the contour model. The foreman video contains rapid movement in few of the frames and hence mean-shift tracker fails to localize the head of foreman and due to clutter background, the result has been degraded in comparison with other videos.

**Table 1:** Result evaluation and accuracy calculation using (%) of correctly tracked frame.

Video	Size	Frame rate	No. of frames	Accuracy (%)	Execution time	Iterations
Table tennis ball	325 × 240	15	52	96.16	250	40
Hand	480 × 640	30	186	91.91	2,100	80
Foreman	192 × 144	30	153	52.94	840	200
Man	380 × 216	30	218	83.50	4,380	320
Akiyo	192 × 144	30	300	68.34	1,020	120

Traditional level-set based active contour model requires a computational expensive re-initialization step to keep the level set function closed to *ϕ*. The use of smoothing and binarization process in the active contour model removed the re-initialization process making the evolution of active contour fast. Also, the proposed active contour is placement insensitive. When mean-shift tracker initialize the contour (i.e. rectangle bounding box) far from the desired object, the evaluation of contour correctly segment the desired object.

Table 2 presents and compares accuracy using correctly tracked frames. Irrespective of dataset, the proposed approach is compared with state-of-art methods of non-rigid object tracking. Most of the state-of-art methods used the VOT2104 dataset (Kristan et al., 2014) consisting of 25 sequences with a total size of more than 10,000 frames. The first comparison is established with Hough transform based pixel voting (PixVot) method which is integrated with probabilistic segmentation algorithm (Duffner & Garcia, 2013) and a patch tracking (PT) method. Region of tracking was divided into patches and correlation filter was used to track in Li, Zhu & Hoi (2015). Sevilla-Lara & Learned-Miller (2012) estimated the distribution field (DF) using features of target objects. Another Hough tracker (HT) based graph cut algorithm was presented in Godec, Roth & Bischof (2013) for non-rigid object tracking. In (Sun et al., 2015), a supervised level-set model (SLM) was proposed where target was tracked using online boosting approach. The mean-shift kernel is sensitive to the orientation and scale of the object. Hence, adaptive tracker driven by data referred to as data-driven kernel (DDK) using level-set was proposed in (Sun et al., 2020). Due to placement insensitive nature of active contour used in the proposed model boosted the performance in comparison with (Sun et al., 2020).

**Table 2:** Comparative analysis of object tracking accuracy using correctly tracked frames.

Method	PixTrac (Duffner & Garcia, 2013)	DF (Sevilla-Lara & Learned-Miller, 2012)	HT (Godec, Roth & Bischof, 2013)	SLM (Sun et al., 2015)	PT (Li, Zhu & Hoi, 2015)	DDK (Sun et al., 2020)	Proposed
Average accuracy	41.33	44.99	51.72	56.41	65.48	65.92	71.86

## Conclusion

In contrast to rectangle boundary representation in visual tracking, this paper proposes the appearance based representation in the tracking process. Simultaneous tracking of an object and its non-rigid shape extraction is a crucial task. This paper modified and proposed an active contour based tracking algorithm. A mean-shift tracker is incorporated in active contour model to track object using feature density function. To reduce the computational complexity, a re-initialization and regularization less active contour model was used in the proposed model. The independent contour deforms according to the shape of the object and hence overcome the weakness of false tracking of mean-shift tracker. The result analysis propagates that the proposed model outperforms other models by increasing accuracy to 71.86%. The proposed model is applicable to single object tracking. The fixed number of iterations have been used in evaluating the contour. This iteration highly depends on the size of the object to be tracked. Therefore, if the size of the object scales down dramatically, then it may not be able to segment the tracked object accurately. Therefore, a stopping criterion to stop contour evaluation is required. A further modification is required to track multiple objects using the active contour model as well as to tackle the occlusion scenario.

## Supplemental Information

10.7717/peerj-cs.373/supp-1 Supplemental Information 1 Illustration of miss-segmentation: Frame: 155 from foreman. Image credit: © iStockvideo.

10.7717/peerj-cs.373/supp-2 Supplemental Information 2 Illustration of miss-segmentation: Frame: 262 from Akiyo.

10.7717/peerj-cs.373/supp-3 Supplemental Information 3 Frame: 74 from man sequences.

10.7717/peerj-cs.373/supp-4 Supplemental Information 4 Tennis Ball Segmentation.

10.7717/peerj-cs.373/supp-5 Supplemental Information 5 New Reader Segmentation.

10.7717/peerj-cs.373/supp-6 Supplemental Information 6 Man tracking and segmentation. Video credit: © iStockvideo.

## Additional Information and Declarations

### Data Availability

The following information was supplied regarding data availability: Sample videos for non-rigid object tracking are available in the Supplemental Files.
