# Supplementary figures and images for: A fast region-based active contour for non-rigid object tracking and its shape retrieval
Source: PeerJ Comput Sci. 2021 May 27;7:e373. doi: 10.7717/peerj-cs.373 (PMC8176551; doi:10.7717/peerj-cs.373)

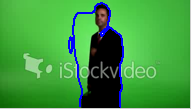

Supplement: Supplemental Information 1 — Image credit: © iStockvideo. [file peerj-cs-07-373-s001.png]

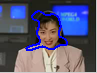

Supplement: Supplemental Information 2 [file peerj-cs-07-373-s002.png]

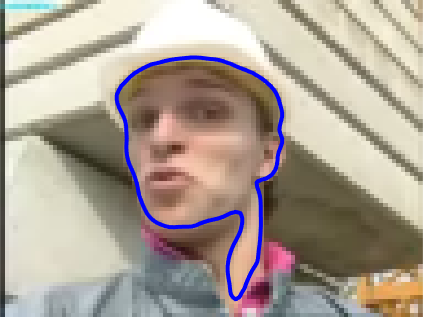

Supplement: Supplemental Information 3 [file peerj-cs-07-373-s003.png]
